# The Broader Autism Phenotype and Its Implications on the Etiology and Treatment of Autism Spectrum Disorders

**DOI:** 10.1155/2011/545901

**Published:** 2011-08-17

**Authors:** Jennifer Gerdts, Raphael Bernier

**Affiliations:** ^1^Department of Psychology, University of Washington, Seattle, WA 98195, USA; ^2^Department of Psychiatry and Behavioral Sciences, University of Washington, Seattle, WA 98195, USA

## Abstract

The presence of autism-related traits has been well documented in undiagnosed family members of individuals with autism spectrum disorder (ASD). The most common finding is mild impairments in social and communication skills that are similar to those shown by individuals with autism, but exhibited to a lesser degree. Termed the *broader autism phenotype* (BAP), these traits suggest a genetic liability for autism-related traits in families. Genetic influence in autism is strong, with identical twins showing high concordance for the diagnosis and related traits and approximately 20% of all ASD cases having an identified genetic mechanism. This paper highlights the studies conducted to date regarding the BAP and considers the implications of these findings for the etiology and treatment of ASD.

## 1. Autism Spectrum Disorders

Autism Spectrum Disorder (ASD), including Autistic Disorder, Asperger's Disorder, and Pervasive Developmental Disorder-Not Otherwise Specified (PDD-NOS), is both genetically and phenotypically heterogeneous. ASD is diagnosed in males four times more often than in females, approximately 65% of individuals with ASD have cooccurring intellectual disability [1], language abilities range from nonverbal to verbally fluent [2], and regression between one and two years of age (mean age 17 months) is reported in approximately one-third of all cases [3]. 

Genetic heterogeneity is noted in ASD as well. Genetic influence in autism is strong, with concordance rates in twins ranging from 60–96% in monozygotic (MZ) twins compared to 0−23% in dizygotic (DZ) twins depending upon the sample and diagnostic boundaries [4–6]. Approximately 10–20% of ASD cases likely have a genetic cause through an identifiable genetic syndrome, observable genetic mutation, or *de novo* copy number variant (CNV; [7]). Nearly 20 mutations relevant in ASD have been identified to date, with more likely on the horizon [7]. However, no single genetic mutation explains a large number of ASD cases and the presence of any one of the risk CNVs does not necessarily prescribe a diagnosis. 

## 2. Broader Autism Phenotype

Psychological characteristics of relatives of children with ASD were first noted in Leo Kanner's 1943 paper in which he noted “For the most part, the parents, grandparents, and collaterals are persons strongly preoccupied with abstractions of a scientific, literary, or artistic nature, and limited in genuine interest in people [44, page 250].” Empirical studies following this line of research initially focused on rates of cognitive disorders, such as intellectual disability, learning disabilities, and language disorders, in family members without a diagnosis of ASD. Elevated rates of such disorders were noted in siblings of individuals with ASD [45, 46]. However, later reports suggested that these cognitive deficits may instead have been markers of intellectual disability rather than autism per se as they were generally present in relatives of individuals with ASD and intellectual disability, but not in relatives of children with ASD with normal intelligence [47]. Since then, the majority of studies have failed to document increased rates of cognitive dysfunction in relatives, regardless of the intelligence level of the affected child [11, 17, 48]. 

Studies do consistently describe a milder phenotype in relatives of individuals with ASD that is qualitatively similar to the defining features within the three domains of ASD: social, communication, and restricted interests and behaviors (e.g., [10, 12, 16, 22]). These subclinical differences in social skills and traits, communication abilities, and personality traits are generally considered to constitute the *broader autism phenotype* (BAP). Studies have generally compared parents of children with ASD (hereafter termed *ASD parents*) and unaffected siblings of individuals with ASD (hereafter termed *ASD siblings*) to family members of control subjects. Subsequent studies have identified variations in social cognition abilities (e.g., [49]), neurocognitive functioning (e.g., [50]), and biological dimensions (e.g., [51]) as well that perhaps relate to or explain the clinical presentation of the BAP. Early markers of the BAP are currently under investigation in infancy (e.g., [52–54]). 

 The purpose of this paper is to provide a comprehensive review of the available literature on the BAP in ASD parents and siblings. We begin by reviewing studies examining social abilities, communication skills, and personality traits. We then summarize measures available to assess the BAP in ASD parents and siblings. In subsequent sections, we explore neurocognitive functioning in relatives as well as studies conducted on biological features, such as head circumference and neural functioning, of ASD parents and siblings. Additionally, we summarize findings on the relationship between characteristics of individuals with ASD, family structure, and the BAP in ASD parents and siblings. Finally, we discuss the clinical and etiological implications of the BAP. 

The BAP itself is not a diagnostic entity. In general, difficulties and differences evidenced in ASD parents and siblings are much milder than those of their child/sibling with ASD and do not fall in the clinically significant range. ASD parents and siblings may demonstrate more BAP features than controls, but the traits, by definition, do not cause enough functional impairment to justify a clinical diagnosis. Therefore, while we summarize a number of statistically significant differences between family members and controls within this line of investigation, we do not suggest that that these features generally warrant clinical concern.

Like the diagnosis of ASD, BAP traits tend to aggregate more often in male relatives than female relatives [12, 16, 19, 30, 31], although this finding is not universal (e.g., [10, 15]). Informant report of social, communication, and repetitive behaviors in grandparents, aunt/uncles, and first cousins of individuals with ASD have suggested that these relatives exhibit similar, but less pronounced differences [16, 19, 55]. Importantly, most studies find that at least half of relatives do not have quantifiable impairments (e.g., [8, 10, 15, 27]), implying that differences may be present in only a subset of family members. 

### 2.1. Social Abilities, Communication Skills, and Personality Traits

The most consistent evidence for the BAP emerged from studies directly assessing social communicative skills, personality traits, and histories in relatives. Early criticisms of such studies centered on the use of relatives of typically developing children as control groups. Critics noted that the differences found in relatives of children with ASD might have resulted from the environmental effects of living with and/or raising a child with a severe disability rather than genetic similarity. Therefore, many researchers changed the nature of their control groups to include family members of children with a developmental disability, often Down syndrome (DS), in order to control for the effects of having a child with disability as a family member. While including families of children with a nonfamilial disability such as DS addresses the environmental influences of living with a child with a significant disability, some researchers have preferred to include a second control group to account for other familial aspects of ASD. For example, developmental language disorders often have a strong genetic component and are included in some studies to control for genetic influences in ASD (e.g., [56]). 

Social traits, such as decreased interest in reciprocal social interactions and a focus on special interests as a conversational topic, identified using clinical interviews have been noted more often in ASD parents and siblings compared to parents and siblings in control groups, [8, 12, 16]. Personality types relating to decreased interest in social interactions (“aloof”), showing a restricted range of affective expression (“undemonstrative”), and using behavior that is interpreted to be off-putting (“untactful”) are also more often more common in ASD parents and siblings compared to controls [13, 15, 18]. Other personality traits (“rigid”) relating to decreased flexibility and difficulty adjusting to changes (e.g., altered routines) have also been reported in ASD parents compared to controls [15, 33]. Self-report generally corroborates findings from clinical evaluations and interviews [20, 21].

ASD parents and siblings also consistently show differences compared to control groups in social communication skills using clinical interview [10, 15, 23, 57]. Self-report of pragmatic language, the social use of language in the context of verbal or nonverbal exchanges, in parents supports these findings [27].  Table 1 provides an overview of the chief findings from BAP studies conducted to date examining social abilities, communication skills, and ASD-related personality traits in ASD parents and siblings.  Table 2 outlines measures designed specifically to assess these traits. 

### 2.2. BAP in Infancy

The majority of BAP studies have focused on parents and school age siblings of children with ASD. However, prospective studies following infant siblings of older children with ASD are ongoing to determine the nature and timing of the BAP in early childhood. Although the precise presentation of the BAP in infancy and its relation to later development remains unclear, several studies have documented social and communication differences in infant/toddler-aged younger siblings of children with ASD who do not go on to be diagnosed ASD (hereafter termed *non-ASD sibs*) (e.g., [58–60]). A study examining the BAP in toddler-aged non-ASD sibs reported decreased social communication, cognitive, adaptive, and language abilities via both observational and parent report measures [58]. The use of pointing, directed facial expressions, and the quality of social overtures discriminated non-ASD sibs from control infant siblings in a different sample at 18 months of age [61]. Hutman and colleagues [62] reported that while response to name and showing appropriate response to distress was impaired in infant siblings who were diagnosed with ASD, these variables did not distinguish non-ASD sibs from typical infants. This suggests that response to name and response to distress are not early signs of the BAP, but may be part of the early presentation of ASD [62]. Gamliel et al. [52] reported that some non-ASD sibs with cognitive delays at 14 and 24 months were no longer delayed cognitively in preschool years. However, expressive and language delays remained in a portion of these non-ASD sibs [52]. The language-related delays continued into school age years and emerged as language-related learning and academic difficulties in 40% of ASD siblings versus 16% of control children, as reported in a follow-up study [63]. Therefore, there may be variability in the developmental trajectory of non-ASD sibs, with delays from some early challenges resolving by later childhood and others continuing into school age years. 

Christensen and colleagues reported fewer functional play skills and increased repetitive nonfunctional play in non-ASD sibs compared to controls [53]. Increased repetitive interests and atypical sensory behaviors were also observed in non-ASD sibs versus typical infants in a separate longitudinal study [61]. A number of studies on infant siblings have reported decreased visual attention to social stimuli, increased attention to nonsocial stimuli, and difficulty disengaging from one stimuli to another [59, 64, 65]. However, diagnostic outcomes have not yet been reported in these studies, so it is unclear if these traits are early markers of ASD itself or an early manifestation of the BAP. 

Overall, ASD-related traits may be present at a very early age in relatives of individuals with ASD; however, followup of these infants and toddlers are necessary as certain findings have been inconsistent across studies and some of these differences have resolved later in life. See Rogers [66] and Zwaigenbaum et al. [54] for more comprehensive reviews of this emerging area of interest. 

## 3. Depression and Anxiety

Piven and colleagues [67] first reported increased rates of depression and anxiety in adult siblings of individuals with ASD based on direct assessments. Additional studies corroborated these early reports, with major depression, obsessive compulsive disorder, and social phobia being the most commonly observed diagnoses in first degree relatives [68–71]. Depression and anxiety diagnoses seem to be more prevalent in female relatives, particularly mothers, compared to male relatives [71]. It is reasonable to assume that having a child with autism significantly impacts mood and anxiety levels. However, studies have indicated that affective episodes in relatives had an onset prior to the birth of their child(ren) with ASD and suggest that the stress of raising a child with autism did not cause the psychopathology [68, 70]. Additionally, mood and anxiety symptoms are largely unrelated to the presence of BAP traits in an individual [68, 70], although a more recent study did report a relationship between depressive symptomotology and BAP traits via self-report on the AQ [72]. 

## 4. Cognition

### 4.1. Intelligence/General Cognitive Abilities

Intelligence and general cognitive abilities in individuals with ASD can range from significantly below average in the intellectual disability range to above average in the intellectually gifted range. Given these variable intelligence levels, researchers have examined intellectual functioning in relatives of children with ASD. Although early reports suggested below average intellectual functioning and cognitive disabilities in ASD siblings [45, 46], many subsequent studies have failed to support these initial findings [11, 17, 48]. Full scale IQ in ASD parents and siblings has since been found to be in the average to high average range in most research studies (e.g., [11, 42, 48, 73–75]). 

Another often-reported finding in individuals with ASD is a pattern of significant variability in cognitive abilities, such as enhanced visual spatial and nonverbal reasoning skills compared to knowledge of vocabulary and comprehension of language [76, 77]. Studies of ASD parents and siblings have similarly documented increased variability in general cognitive abilities as measured by intelligence tests compared to control groups suggesting a possible association of cognitive variability to the BAP [17, 56, 73, 78, 79]. 

### 4.2. Central Coherence

There are several cognitive theories that attempt to explain the triad of impairments in ASD. One such theory involves a cognitive process called *central coherence*. Central coherence is a model of cognitive processing style ranging from a tendency to focus on smaller details with a weakness in seeing more global perspectives (local processing bias) to a propensity for seeing the gestalt while sacrificing details (global processing bias). Weak central coherence results in an overfocus on the parts rather than the whole while strong central coherence involves a good understanding of the larger picture, but less attention to details. The block design subtest included in many intelligence tests has been considered a proxy for central coherence since breaking down the picture to be replicated into its parts is the most efficient strategy for completing the task. Individuals with ASD have been purported by some groups to have weak central coherence [80, 81] while others have reported intact central coherence in children with ASD [82–84]. Thus, the validity of the central coherence theory in ASD remains unclear. 

In a two-part exploration of central coherence in relatives, Briskman and colleagues [20, 85] compared central coherence among parents and siblings of individuals in three groups: no developmental abnormality, dyslexia, and ASD. Fathers of children with ASD demonstrated differential performance suggestive of weak central coherence on all measures [85]. There were no differences between mothers of children with ASD and controls on any of the tasks, suggesting sex differences in central coherence in relatives. On self-report assessing the social and nonsocial (e.g., special interests, sensory sensitivities, and preference for routines) aspects of the BAP, those parents (particularly fathers) with greater nonsocial scores tended to have weaker central coherence [85]. A separate research group has also documented weak central coherence compared to controls [49]. 

However, weak central coherence was reported to be absent in ASD siblings, suggesting no apparent differences in central coherence in ASD siblings, male or female [20]. A number of additional studies have reported intact central coherence and failed to document differences between ASD relatives and controls in tasks purported to tap into central coherence, such as block design [28, 29, 78, 86]. Therefore, like the debate about the validity of the central coherence theory in individuals with ASD, the relation of weak central coherence to the BAP remains to be determined. 

### 4.3. Executive Functioning

Another cognitive theory of the underlying processing mechanism in ASD involves a primary deficit in executive functioning (EF). EF encompasses abilities that underlie goal-directed behavior, including working memory, inhibition, cognitive flexibility, and planning. Individuals with ASD often have difficulty with EF tasks [87, 88]. However, there is substantial debate over the exact nature of EF challenges in ASD, and differences noted may be measure specific. 

Differences in EF are documented in ASD parents and siblings as well compared to controls. Compared to parents of children with learning disabilities and typically developing children, ASD parents, particularly fathers, showed challenges on computerized tasks of four components of EF: attentional flexibility, planning, spatial working memory, and spatial short-term memory [89]. Attentional flexibility was particularly difficult with half of ASD parents showing an impaired ability to shift problem-solving strategies when necessary compared to a small percentage of control parents. In another study, parents within multiple-incidence ASD families demonstrated poorer performance compared to parents of children with DS on the Tower of Hanoi task, a measure of planning [78].

Studies of ASD siblings have showed the same general trend. Hughes and colleagues [50] found that a greater percentage of ASD siblings demonstrated increased difficulty with attentional flexibility and more advanced stages of planning on a computerized EF task compared to siblings of children with severe to moderate developmental delays and neurotypical children. In another study, there was a significant difference between ASD siblings' performance on the Tower of Hanoi task compared to siblings of children with various other learning disabilities [90]. Precursors to executive functioning abilities have recently been examined in infancy. Latency in disengaging from one stimulus to attend to another has been reported in a subset of infant siblings of children with ASD compared to control infants [91]. 

Wong and colleagues [92] suggest that poor planning may reflect an EF challenge in generativity in ASD, meaning difficulty generating more than one strategy to solve a problem (also called *fluency*). In their study comparing ASD parents and siblings to relatives of children with mild intellectual disabilities, they found differences in several measures of verbal and nonverbal generativity/fluency between index family members and controls [92]. 

Working memory challenges that are often present in individuals with ASD are also present to a lesser degree in ASD parents. One study reported that ASD parents scored significantly lower on a verbal working memory task compared to parents of typically developing children [74]. ASD parents made more errors in a delayed oculomotor response task assessing spatial working memory via tracking of saccades (or fast movements of the eye) compared to control adults [93]. However, many of these same EF studies have not documented differences in every measure in their battery [11, 50, 74, 92, 94] and some have found no differences on any measure of EF [29]. 

### 4.4. Social Cognition

An additional theory purporting to explain the underlying social and communication difficulties observed in ASD involves a primary deficit in social cognition: specifically a construct called *theory of mind *(ToM). ToM involves the ability to understand other's emotions, motivations, and intent. It allows individuals to take another person's perspective and intuit his or her mental state.

Mild ToM challenges in using facial cues and other features to determine mental states have been noted in relatives of individuals with ASD. For instance, Baron-Cohen and Hammer [49] found that ASD parents, particularly fathers, were slightly less able to determine the thoughts and feelings of people based solely on photographs of their eyes (*Reading the Mind from the Eyes* test) compared to adult controls. Another study using this task found a trend toward differences in ASD parents compared to parents of typically developing children [74]; a similar pattern of results has been noted in ASD siblings compared to control children using the same measure [95]. Significant differences in an advanced ToM task were detected in ASD parents' ability to reason about emotions in order to correct inconsistencies in emotional expressions. Additional studies have reported similar differences using advanced ToM experimental tasks [23] and self-report of emotion processing abilities, particularly emotion identification [96], in ASD parents compared to control parents. However, several studies have reported no difficulties in ToM tasks in ASD siblings compared to siblings of individuals with other developmental disabilities [11, 90, 97].

The relationship between social and communication skills and ToM abilities in ASD parents has also been examined. Findings from two recent studies suggest that impairments on specific aspects of social cognition may only be relevant for subgroups of ASD parents with social features of the BAP: namely, those with “aloof” personality styles involving social dispositions relating to preference for alone time and infrequent use of social chat during social exchanges [25, 29]. ASD parents without this personality style did not demonstrate difficulties on ToM tasks. 

## 5. Language Abilities

### 5.1. Phonological Processing and Reading Abilities

Phonological processing is the manner by which written and spoken words are processed and can be crucial for strong reading and writing abilities. These skills are heritable (e.g., [98, 99]) and some children with ASD show marked impairments in measures of phonological processing [100]. Evidence suggests that only children with ASD who have a language impairment (and not those without a language impairment) have challenges in phonological processing and reading-related skills, similar to children with a Specific Language Impairment (SLI) without ASD [100–103]. Therefore, it is possible that reading-related challenges and phonological processing skills are not specific to the ASD phenotype per se, but related to overall language challenges. 

Compared to age- and sex-matched adults, ASD parents scored lower on the nonword repetition task (a measure of phonological processing; [79]). This was a particular challenge for nonwords of three syllables or more, suggesting that phonological short-term memory, rather than perception of sounds or sound production, may be a specific area of interest for future study. A similar report of increased difficulties in reading of nonwords longer than three syllables in a much larger sample was noted in ASD parents (particularly those with a history of language-related challenges) compared to DS parents [17]. In a study of multiplex families, ASD parents demonstrated weaker performance on some reading measures (e.g., passage comprehension and rapid automatized naming) compared to parents of individuals with DS [78]. Rapid automatized naming skills in ASD parents and their high-functioning children were related, with both groups showing longer latencies than DS parents [104]. Rapid automatized naming skills in ASD parents (particularly fathers) related to retrospective reports of early language delay and were associated with a socially reticent personality style. 

However, many studies have failed to report difficulties in phonological processing and reading abilities in relatives. For example, Bishop and colleagues [105] found no differences between parents and siblings of children with ASD versus those of typically developing children of a variety of different IQ levels on measures of phonological processing (nonword repetition and reading of nonword passages). Additionally, no differences between ASD siblings and control siblings were noted in other studies examining word fluency, rapid automatized naming, and pragmatic language [56, 97]. An additional study compared language, reading, and phonological processing abilities in ASD parents and siblings of children both with and without language impairment to parents and siblings of children with SLI [101]. While parents and siblings of children with ASD who had a language impairment scored lower than relatives of children with ASD who did not have an impairment, neither groups' scores were in the impaired range. Additionally, SLI relatives scored significantly lower than both groups, suggesting separate genetic etiologies of language impairments in probands within these two disorders [101]. Therefore, research suggests that challenges in reading abilities and phonological processing may be absent in ASD siblings and unclear in ASD parents. 

### 5.2. Structural Language

ASD parents and siblings are more likely than controls to have had an expressive language delay in childhood as well as other language-based learning deficits, such as dyslexia [12, 14, 16, 106]. However, a number of other studies have failed to report difficulties in structural language problems in relatives. Whitehouse et al. [27] compared ASD parents to parents of children with language and/or literacy impairments and typically developing children found that structural language-related challenges only presented in parents of children with language/literacy impairments, but not those of children with ASD. In ASD siblings, Pilowsky and colleagues [56] found that ASD siblings and siblings of children with intellectual disability had better developed receptive and expressive language skills and higher verbal IQs than siblings of children with language disorders [56]. Therefore, it is unclear the extent of these reported language difficulties and it is possible that some early language challenges may resolve by later childhood and adulthood. 

## 6. Biological Dimensions

### 6.1. Head Circumference and Brain Volume

Macrocephaly is a consistent physical finding reported in individuals with ASD. Indeed, many studies have found increased head circumference (HC) in both children and adults with ASD compared to age-based normative measurements [5, 107–110]. Similarly, magnetic resonance imaging (MRI) studies find increased cerebral volume in children with ASD compared to typically developing children and those with other developmental delays [111, 112]. Brain overgrowth appears to represent both greater brain tissue volume and greater lateral ventricle volume [112].

Despite this well-replicated finding of increased head size and brain volume in ASD, macrocephaly does not appear to define a specific subgroup of clinical features. Some studies have found that increased head size in ASD is advantageous and is related to higher IQ [113] and better social skills [109] whereas others have reported negative effects, such as delayed onset of words [110], more impaired social cognition [113], and increased stereotyped behaviors [114]. Still other studies have found no direct associations between HC-and ASD-related symptoms [108]. Children later diagnosed with more severe ASD demonstrated a faster and greater rate of brain overgrowth during infancy, compared to children with less severe symptoms [107]. 

Less research has been conducted on HC/brain volume among family members of individuals with ASD. The limited research available does suggest that head size is increased in family members. In first-degree relatives, 18.9% of ASD parents and 11.4% of ASD siblings had HC measurements exceeding the 97th percentile [114]. These numbers were significantly elevated compared to normative measurements, but were not different from the comparison group, consisting of family members of individuals with tuberous sclerosis and seizure disorders. In other family samples, HC of ASD parents were skewed toward macrocephaly [110, 115]. The trait appears to be familial as 35–45% of affected children with macrocephaly had a macrocephalic parent [110, 115]. However, in another sample, brain volume of ASD parents was not different from controls [116]. 

Two studies have examined the relation between HC and BAP traits in ASD relatives. Elder and colleagues [51] reported that young siblings of children with ASD with a faster rate of growth in HC in the first year of life followed by slowing of growth in the next year had a greater number of BAP traits. In contrast, Constantino and colleagues [117] failed to find a relationship between ASD traits as measured by the SRS and rate of head growth in infant male siblings of children with ASD. 

### 6.2. Neural Functioning and Structure

Neural studies using technologies such as electroencephalography (EEG) and functional MRI (fMRI) provide further support for biological differences between ASD parents and siblings and control groups. In a small pilot study, Baron-Cohen and colleagues [118] compared ASD parents to adult controls using fMRI on a visual search task and an advanced emotion recognition task (both of which differentiate controls from individuals with ASD using the same technology). Findings corroborated an earlier report from the same group of differences in behavioral outcomes on both of these tasks [49] as well as atypical brain response compared to controls. Additionally, mothers and fathers processed the tasks differently at a neural level, providing further support for sex differences in the presence of the BAP [118].

Facial processing also appears to be different in ASD parents and siblings. Dawson and colleagues [119] reported that ASD parents showed a delay in the face-related event related potential (ERP) component, the N170, when looking at faces, although not when looking at objects, suggesting a neural difference in face processing [119]. In a second facial processing study using eye tracking and imaging, ASD siblings spent significantly less time looking at eyes in photographs compared to controls, but spent the same amount of time looking at eyes as their siblings with ASD [120]. The ASD siblings showed greater activation to faces in the right posterior fusiform gyrus but not the left, which was largely accounted for by eye fixation [120]. Emerging evidence for an atypical neural response to direct eye gaze in infant siblings has also been reported [121].

Total brain and brain structure volume has been explored in ASD parents and ASD siblings. Kates and colleagues [122] found that monozygotic twin pairs who were concordant or discordant for ASD had similar cerebral gray and white matter volumes. However, only the concordant twin pairs had similar *cerebellar* gray and white matter volumes. Compared to control subjects, both diagnosed and undiagnosed twins within discordant twin pairs exhibited lower frontal, temporal, and occipital white matter volumes, suggesting that this neural difference is also present in undiagnosed relatives [122]. Compared to controls, ASD siblings show decreased amygdala volume [120]. Conversely, in ASD parents, left hippocampal volume was found to be larger compared to typical adults [123]. ASD parents have also shown increases and decreases in regional gray matter volume using voxel-based morphometry, a finding similar to observed variations in brain structure in individuals with ASD [124]. 

## 7. Moderators of the BAP

### 7.1. Traits of Affected Children

Various aspects of the BAP appear to be present in relatives across subtypes of individuals with ASD. ASD parents whose children experience a regression in skills demonstrate characteristics of the BAP at the same rates as those whose children do not experience a regression (27.8% versus 32.9%; [75]). Child gender also does not appear to be related to the degree of expression of the BAP in relatives [12, 55]. Cognitive ability of children with ASD is unrelated to the degree of expression of the BAP in parents [13, 17, 21, 48]. However, Szatmari and colleagues [55] reported that social and communication impairments were more common in immediate and extended relatives of affected children with IQs above versus below 60. 

The severity of ASD symptoms in affected children appears to be related to BAP traits in family members. Pickles and colleagues [19] found that the severity of symptoms in verbal children with ASD was related to the degree of expression of the BAP in family members, but was unrelated in nonverbal affected children. Similarly, self- and informant-reports of autism-related traits in the typical population indicate that families in which both parents show subthreshold social reciprocity challenges are more likely to have children with scores in the impaired range of social behavior suggestive of an ASD diagnosis [125]. Additionally, while social responsiveness (assessed via the SRS) in parents, probands, and siblings did not predict *diagnostic status* of infant siblings in a one study, social responsiveness in fathers predicted proband social responsiveness and proband responsiveness predicted sibling responsiveness [30]. Therefore, there is some evidence that the severity of autism-related traits is correlated within some families, particularly in males. 

### 7.2. Family Structure

The number of affected children within a family also relates to BAP traits in family members. A number of studies to date have compared relatives within simplex and multiplex ASD families on measures of the BAP. Szatmari and colleagues [55] screened nearly 2,000 immediate and extended relatives and found that social impairments, but not communication challenges or presence of restricted behaviors, were more common in multiplex relatives as compared to simplex relatives [55]. Bölte and Poustka [126] found that simplex ASD parents and siblings demonstrated superior performance in emotion recognition compared to ASD parents and siblings within multiplex families. Virkud and colleagues [127] found that mean scores on the Social Responsiveness Scales (SRS) in ASD male siblings from simplex families were substantially lower than mean scores in ASD siblings from multiplex families. There was a trend in the same direction on spouse-report SRS for fathers, but not mothers [127]. A more recent study examining relatives within multiplex and simplex ASD families and relatives of typically developing children replicated findings of increased BAP severity in siblings [30]. Siblings within multiplex families had significantly greater SRS scores than siblings within simplex families, with both groups showing greater BAP severity than typical siblings. No differences between multiplex and simplex fathers or mothers were reported [30].

In the most extensively phenotyped sample to date, Losh and colleagues [128] found a nearly consistent linear trend across measures of personality traits associated with the BAP, friendship preferences, and pragmatic language between simplex and multiplex ASD parents and DS parents. The multiplex ASD parents had more BAP traits than simplex ASD parents and the simplex ASD parents had more traits than DS parents. Additionally, it was more common in multiplex than simplex ASD families for both parents to show features of the BAP [128]. Schwichtenberg and colleagues [30] prospectively followed infant siblings of children with ASD and found that infants within multiplex families were more likely (64%) to develop ASD than simplex (9%) and control families (4%). 

Overall, these findings lend support to the theory that families containing multiple children with ASD families carry a higher loading for ASD-related traits given that the presence of such traits is more common in this family type than in families containing just one child with ASD. Additionally, a number of genetic studies have found that *de novo* or sporadic CNVs (a type of genetic mutation) are more common in simplex ASD families as compared to both multiplex ASD families and families without any history of ASD [129–131]. Therefore, it is possible that the types of genetic causes of ASD may vary between single-incidence and multiple-incidence families.

If affected children from simplex families are more likely than those from multiplex families to develop ASD as a result of a *de novo* genetic event occurring only in that individual, then findings of an increased presence of ASD-related traits in multiplex families may suggest that such family members are more vulnerable to ASD symptoms given shared genetic variance. However, the validity of this phenomenon requires future research attention since few studies have directly tested this hypothesis and findings are not universal [96, 132]. 

## 8. Summary

A number of domains have been studied in ASD parents and siblings. Mild differences in social and communication skills related to the core deficits in ASD are present in a subset of relatives and are reported nearly consistently across studies. Therefore, social and communication impairments are likely the most familial of autism-related traits. Variable cognitive abilities, differences in theory of mind and executive functioning skills, increased head circumference, and differences in neural functioning and structure have all been documented in ASD parents and siblings. However, many studies have reported contradictory findings in other areas (e.g., central coherence and phonological processing), making their association with the BAP questionable. 

## 9. Etiological and Clinical Implications

The range of potential symptom profiles that can emerge from the varied deficits in social communication and behavior in ASD results in a complex behavioral presentation. The use of general, overarching diagnostic categories in complex psychiatric disorders such as ASD has often muddied genetic studies with these groups. Therefore, defining quantifiable components of the phenotype that are theoretically more closely tied to genetic vulnerability than a qualitative diagnosis helps to isolate traits for genetic and neurobiological analysis [133–135]. Investigations of component traits in biological relatives can also broaden our understanding of the complex systems that underlie social communicative behavior and offer insight into how these systems go awry in ASD. An interactive model of genetic, neurobiological, environmental, and protective factors may explain why significant deficits are present in affected children while only mild challenges are measurable in relatives. In depth examinations of the familiality of component traits provide a more complete picture of ASD's etiology.

Examinations of BAP profiles in families provides an opportunity to gain insight into how phenotype profiles may be inherited within families and informs understanding of varying underlying genetic mechanisms in particular families. For instance, particular BAP profiles may be more suggestive of chromosomal variations impacting specific genomic regions. Specifying particular family types using behavioral measures may allow for the identification of differing genetic mechanisms. Examination of carefully defined phenotypes is crucial in providing insight into behavioral manifestations of genetic events and broadening understanding of the etiology of complex disorders such as ASD.

Consideration of BAP traits in family members also has important clinical implications in terms of treatment planning. An understanding and awareness of parent/familial factors is crucial to the development of any intervention plan with a family seeking treatment for their child. Clinicians often individualize interventions for different children with the same diagnosis based on certain family characteristics and needs. Such family factors can include addressing specific treatment goals that parents have for their child, parent/child motivation for change, varying personality styles, skill level in implementing the intervention, and home and school environments. In ASD, awareness that BAP traits and certain cognitive features are present in some family members should be part of these overall family considerations. 

For example, it is conceivable that a parent with decreased social motivation may perceive his or her child's limited social motivation differently from a parent in a different family who considers an extensive social network essential. From the first parent's perspective, desired treatment goals may deemphasize social engagement relative to other presenting problems while a parent in the latter family may consider goals around social interaction more important than interventions focused on other behaviors. Conversely, it is also possible that parents with decreased social motivation may consider the focus on social engagement in the treatment of their child to be paramount depending specific experiences surrounding their own level of social engagement. Therefore, it may not be the presence of BAP traits per se, but parents' experience and perception of that trait in themselves and their child that may influence treatment planning. Thus, considerations of the BAP in clinical settings may be helpful in recommending and implementing the best and most appropriate treatments for a child. 

The BAP has been used to help elucidate the genetic mechanisms in ASD and provide a clearer picture of the clinical phenotype of ASD. Although further work is certainly needed before the BAP can be fully incorporated into treatment planning, there have been significant advances in our understanding of and ability to assess the BAP. Clearly, these advances in our understanding of the BAP have set the stage for future clinical and scientific pursuits. 

## Figures and Tables

**Table 1:** Investigations of social abilities, communication skills, and personality traits in ASD parents and siblings.

	Sample	Control group	Measures of social communication skills	Key findings
Wolff et al. 1988 [8]	(i) Affected children all had useful language (ii) ASD parents (21 mothers, 14 fathers) (iii) Control Parents (21 mothers, 18 fathers)	Parents of children with variety of developmental delays, including DS, Prader-Willi Syndrome, and idiopathic delays	Personality interview developed by author [9]	(i) 46% of ASD parents, particularly fathers, versus 0% of controls had “schizoid” personality traits, meaning impaired rapport with interviewers, suspiciousness, low-emotional responsiveness, difficulties with communication (either over or under), guardedness, and excessive discussion of special interests (ii) Most ASD parents did not meet full criteria for a personality disorder

Landa et al. 1992 [10]	(i) 43 ASD parents (sex breakdown not reported) (ii) 21 Control Adults (sex breakdown not reported)	DS parents and adults with no children with ASD	PRS	(i) Using blindly rated scores, 42% of ASD parents had some pragmatic language deficit compared to 2% of controls (ii) No sex differences and scores were not significantly correlated with IQ

Szatmari et al. 1993 [11]	(i) Affected children had a variety of cognitive levels, evenly sampled across severity levels (*IQ* < 50, 51–70, and 70+) (ii) ASD parents (51 mothers, 46 fathers) (iii) Control Parents (30 mothers, 24 fathers) (iv) 72 ASD siblings (aged 6–18 years) (v) 46 Control siblings (aged 6–18 years)	DS parents (to control for low IQs) and parents of very low birth weight children (to control for higher IQs)	(i) Relative's Screening Interview (developmental history) (ii) VABS	(i) Primarily examined cognitive functioning and results are described in text (ii) No differences on the social and communication domains of the VABS in ASD siblings compared to control siblings or across IQ of the affected child (all were in average range) (iii) No differences per developmental history of social and communication delays between the groups (i.e., 18.8% of ASD siblings had social problems compared to 15.9% of control siblings)

Bolton et al. 1994 [12]	(i) Affected children were oversampled for females. Sampled evenly for IQs between 30–49, 50–69, and 70+. (ii) ASD Relatives (198 parents, 137 siblings) (iii) Control Relatives (72 parents, 64 siblings)	DS parents and siblings	FHI	(i) 20.4% of ASD siblings demonstrated either communication atypicalities, social impairments, or restricted behaviors compared to 3.1% of control siblings (ii) Parents showed this same pattern of results, to a lesser degree (iii) More common among males (iv) Severity of *lesser variant *was related to severity of symptoms in child with ASD only for those children with speech
	(i) Affected children had IQs evenly distributed across severity levels.			(i) 24% of ASD parents were rated to be aloof (decreased interest in social interaction) compared to 8% of DS parents
	(ii) ASD parents (45 mothers, 42 fathers)	DS parents	M-PAS	(ii) 22% of ASD parents were rated to be undemonstrative (restricted range of affective expression) compared to 11% of DS parents
Piven et al. 1994 [13]	(iii) Control Parents (19 mothers, 19 fathers)			(iii) 10% of ASD parents were rated to be untactful (behavior that was interpreted to be off-putting) compared to 3% of DS parents
				(iv) 16% of ASD parents were rated for at least 2 of these traits compared to 0% DS parents
				(v) IQ of children was unrelated to M-PAS ratings in parents

Fombonne et al. 1997 [14]	(i) Same ASD sample as Bolton et al., 1994 [12] (ii) ASD parents (74 fathers, 86 mothers) (iii) Control Parents (19 fathers, 23 mothers) (iv) ASD siblings (62 males, 58 females) (v) Control siblings (16 males, 23 females)	DS relatives	FHI	(i) Primarily examined cognitive functioning in family members and results are described elsewhere (ii) BAP traits were unrelated to IQ scores

Piven et al. 1997 [15]	(i) Multiplex ASD families, 51% of affected children had IQs over 70 (ii) ASD parents (25 mothers, 23 fathers) (iii) Control Parents (30 mothers, 30 fathers)	DS parents	(i) M-PAS-R (ii) PRS	(i) 25–50% of ASD parents showed aloof, hypersensitive to criticism, anxious, and rigidity (difficulty adjusting to change) traits compared to 3–5% of DS parents (ii) No consistent evidence for sex differences (iii) Quality and quantity of friendships was rated lower in ASD parents (particularly fathers) (iv) Significantly more ASD parents had some measure of pragmatic language deficits compared to controls (impairments in 18–25% of ASD parents versus 0–6% of DS parents) (v) Unrelated to IQ scores

Piven et al. 1997 [16]	(i) Same ASD and parent sample as Piven, Palmer, Landa et al., 1997 [15] (ii) 12 ASD siblings (iii) 53 Control siblings (iv) ASD Extended Family Members (96 grandparents, 145 aunts/uncles) (v) Control Extended Family Members (120 grandparents, 168 aunts/uncles)	DS relatives	FHI	(i) Social deficits : 57% of ASD fathers versus 13% of DS fathers; 36% of ASD mothers versus 13% of DS mothers (ii) Communication deficits: 20% of ASD mothers versus 0% of DS mothers; nonsignificant difference in fathers (iii) Stereotyped behaviors: 26% of ASD fathers versus 3% of DS fathers; 12% of ASD mothers versus 0% of DS mothers (iv) ASD siblings and extended relatives demonstrated more social deficits and restricted/repetitive interests on the FHI than controls (more often in male relatives); differences were less pronounced than parents
Folstein et al., 1994 [17]	(i) Affected children stratified to include ~equal numbers across IQ severity levels (<30, 30–50, 50–70, and 70+) (ii) 166 ASD parents (iii) 75 Control Parents (iv) ASD siblings (42 males, 45 females) (v) Control siblings (28 males, 36 females)	DS relatives	(i) FHI (ii) PRS (iii) Friendship Interview (iv) PAS (“Aloof”)	(i) Primarily examined cognitive functioning and results are described elsewhere (ii) ASD parents with early cognitive difficulties on the FHI (e.g., late onset of speech, reading/spelling difficulties) showed greater pragmatic language deficits despite average Verbal IQ compared to control parents (iii) This relationship was insignificant on the “aloof” item of the PAS and the friendship interview

Murphy et al., 2000 [18]	(i) Same ASD sample as Bolton et al., 1994 [12] (ii) ASD Relatives over 18 years old (195 parents and 97 siblings) (iii) Control Relatives over 18 years old (72 parents and 52 siblings)	DS relatives	(i) M-PAS (ii) FHI	(i) In ASD parents, the traits anxious and conscientious were prominent compared to DS Parents (ii) In ASD adult siblings, the traits aloof, shy, undemonstrative, impulsive, sensitive, self-conscious and eccentric were significantly elevated compared to DS adult siblings (iii) Factor analysis revealed three factors on the M-PAS: “Withdrawn,” “Tense,” and “Difficult”. In ASD parents and siblings, the item total for each factor was more than twice that of DS parents and siblings (iv) One SD increase in symptom severity of the affected child on the ADI led to a 17% increase in “withdrawn” factor on M-PAS

Pickles et al., 2000 [19]	(i) Combined two study samples to allow for even distribution of IQ of the affected child (including an oversampling of IQs < 50) (ii) 285 ASD parents (iii) 72 Control Parents (iv) 189 ASD siblings (also 30 half siblings) (v) 64 Control siblings (also 1 half sibling) (vi) ASD Extended Family Members (527 grandparents, 543 aunts/uncles, 774 first cousins) (vii) Control Extended Family Members (139 grandparents, 166 aunts/uncles, 277 first cousins)	DS relatives	FHI	(i) 7.5% of all ASD relatives were classified as BAP by the FHI compared to 2.7% of controls (ii) Differences in extended relatives were less significant than in ASD parents and siblings (iii) More often in males (iv) No evidence for simple X-linked or imprinted X-linked inheritance patterns (v) Severity of BAP traits on the FHI was related to severity of symptoms in the affected child only for those affected children with speech

Briskman et al. 2001 [20]	(i) Affected children had IQ > 65 and were only male (ii) ASD parents (21 mothers, 21 fathers) (iii) Dyslexia Parents (14 mothers, 13 fathers) (iv) Control Parents (14 mothers, 14 fathers) (v) 19 ASD siblings (males only) (vi) 13 Dyslexia siblings (vii) 20 Control siblings	(i) Relatives of individuals with Dyslexia (ii) Relatives without any family members with developmental disorders	(i) Interview developed by authors (i) Parent or self-report of social functioning and nonsocial features related to ASD	(i) ASD parents (particularly fathers) had significantly higher scores than parents in both the dyslexia and typically developing groups (ii) 62% of ASD fathers obtained high total scores compared to 15% of the dyslexia and 0% of the control fathers (iii) Differences not found in siblings
Bishop et al., 2004 [21]	(i) Affected children (59 met for autism, 21 met for PDD-NOS) (ii) ASD parents (65 mothers, 46 fathers) (iii) Control Parents (48 mothers, 37 fathers)	Parents of children without ASD	AQ	(i) Social and communication skills significantly lower in ASD parents (particularly fathers) compared to control parents (ii) Scores on other categories (attention to detail, attention switching, and imagination) did not differ between groups

Bishop et al. 2006 [22]	(i) Same ASD sample as Bishop et al., 2004 [21] (ii) 43 ASD siblings (iii) 46 Control children	Typically developing children of a variety of IQ levels	CCC-2	(i) 23.8% of ASD siblings scored 2 SD below the control mean compared to 2.2% of controls (ii) ASD siblings with low scores on the CCC-2 tended to have fathers with evidence for the BAP via self-report on the AQ (iii) VIQ of the affected child was unrelated to CCC-2 scores in ASD siblings (iv) Differences in structural language skills also noted

Di Michele et al. 2007 [23]	(i) Average FSIQ of affected children was 88.9 (ii) ASD parents (12 mothers, 11 fathers) (iii) DS Parents (10 mothers, 2 fathers) (iv) Control Parents (9 mothers, 14 fathers)	(i) Parents of “healthy” children matched for mental (but not chronological) age of affected child (ii) Parents of children with DS	(i) Gricean conversational maxims tasks (ii) Methods described elsewhere [24]	(i) ASD parents detected fewer pragmatic language errors (2 SD below the norm) in recorded conversations (e.g., failing to perceive redundant, irrelevant, or uninformative information) compared to parents in both control groups

Losh & Piven, 2007 [25]	(i) Affected children were “high functioning” and were either simplex or multiplex (ii) ASD parents (enriched for those with BAP traits as measured by M-PAS-R; 23 fathers, 25 mothers) (iii) Control Parents (9 fathers, 13 mothers)	DS parent or parent of typically developing children	(i) M-PAS-R (ii) PRS (iii) Friendship Interview (iv) Reading the Mind from the Eyes test	(i) Only those parents with aloof personalities had significant difficulty recognizing emotion/mental states (ii) Performance predicted difficulties in pragmatic language and lower quality of friendships for aloof parents only (iii) Offers evidence for distinct subtypes of ASD relatives

Ruser et al., 2007 [26]	(i) Affected children had “sufficient language to complete battery”; average FSIQ was 86.63 (ii) ASD parents (23 fathers, 24 mothers) (iii) SLI Parents (21 fathers, 26 mothers) (iv) DS Parents (10 fathers, 11 mothers)	(i) SLI parents (score <13th percentile on standardized language test or <9th percentile on nonword repetition) (ii) DS parents	PRS-M	(i) ~ 15% of both SLI and ASD parents (particularly fathers) had significant conversation challenges compared to < 5% of DS parents (ii) No significant differences in overall score between SLI and ASD parents on the PRS-M

Whitehouse et al. 2007 [27]	(i) Affected children had NVIQ >85 (ii) ASD parents (20 mothers, 10 fathers) (iii) SLI Parents (22 mothers, 8 fathers) (iv) Typical Parents (23 mothers, 7 fathers)	(i) SLI parents (scores <10th percentile on at least 2 standardized language tests) (ii) Parents of typically developing children	AQ	(i) Primarily examined structural language functioning and results are described elsewhere (ii) Self-report of pragmatic language abilities differentiated SLI from ASD parents on the AQ (iii) 20% of ASD parents showed BAP compared to 3.3% of SLI parents
Scheeren & Stauder, 2008 [28]	(i) Affected children had FSIQ >70 (ii) ASD parents (12 mothers, 13 fathers) (iii) Control Parents (12 mothers, 13 fathers)	Parents of typically developing child without family history of ASD	AQ	(i) No group differences (except control mothers scored higher than ASD mothers on one subtest “attention to detail”)

Losh et al., 2009 [29]	(i) Affected children were >16 years and had NVIQ >80 (ii) 83 ASD parents (iii) 32 Control Parents	Parents of typically developing child without family history of ASD	(i) M-PAS-R (ii) Several social cognition tasks	(i) 27% of ASD parents had social features of BAP (more often in fathers) while 41% had rigidity traits on the M-PAS-R (equal % mothers and fathers) (ii) Only the group of ASD parents with social features of the BAP were less accurate in several social cognition tasks (iii) ASD parents without the social BAP performed similarly to controls on these tasks

Schwichten-berg, et al., 2010 [30]	(i) Affected children diagnosed with autism, Asperger's, or PDD-NOS via ADOS and clinical evaluation (either simplex or multiplex) (ii) All families included mother, father, “proband,” and infant sibling (portion of families had an additional sibling) (iii) 124 Simplex ASD Families (iv) 11 Multiplex ASD Families (v) 82 ASD Controls	(i) Parents, infant, and additional siblings of typically developing child	SRS	(i) At 36 months, infant siblings were categorized as typical, ASD, or “other developmental concerns” (ii) Greater BAP traits in ASD fathers and additional siblings (but not mothers) compared to control fathers (no differences between ASD simplex and multiplex fathers, but multiplex siblings had greater SRS scores than simplex siblings) (iii) ASD multiplex infant siblings more likely (64%) to develop ASD than ASD simplex (9%) and control (4%) (iv) 27% of ASD simplex infant siblings had other developmental concerns versus 11% of typical infant siblings (v) Neither parent, proband, or additional sibling severity predicted infant sibling diagnostic status (vi) Father (but not mother) severity predicted proband severity and proband severity predicted additional sibling severity

Wheelwright et al., 2010 [31]	(i) Affected children (parent-report of diagnosis on the autism spectrum) (ii) 90% simplex, 10% multiplex (iii) ASD Parents (1429 mothers, 571 fathers) (iv) Control Parents (558 mothers, 349 fathers)	Parents of typically developing children	AQ	(i) ASD parents had greater AQ scores in 4 of 5 domains than control parents, with males overall showing greater impairment than females in both groups (ii) 33% of ASD fathers and 23% of ASD mothers versus 22% control fathers and 9% of control mothers had AQ scores at least 1 SD above mean

Abbreviations: ADOS : Autism Diagnostic Observation Schedule; AQ : Autism Spectrum Quotient; ASD parents : undiagnosed parents of individuals diagnosed with ASD; ASD siblings : undiagnosed siblings of individuals diagnosed with ASD; CCC-2 : Children's Communication Checklist-2; DS : Down syndrome; FHI : Family History Interview; M-PAS : Modified Personality Assessment Schedule, M-PAS-R : Modified Personality Assessment Schedule, Revised; PAS : Personality Assessment Schedule; PRS : Pragmatic Rating Scale; S : D Standard Deviation; SLI : Specific Language Impairment; VABS : Vineland Adaptive Behavior Scales.

**Table 2:** Measures developed and utilized to assess the BAP.

	Measure name	Reference	Intended population	Measure notes
	Autism-Spectrum Quotient (AQ)	Baron-Cohen et al. 2001 [32]	Typical adults and ASD parents	(i) A brief, self-report questionnaire assessing 5 domains: social skills, communication, attention to detail, attention switching, and imagination (ii) Differentiates high-functioning individuals with ASD from controls, males from females, and college students majoring in mathematics versus humanities (iii) Identifies higher rates of BAP in ASD parents compared to controls
Questionnaires	Broader Autism Phenotype Questionnaire (BAPQ)	Hurley et al. 2007 [33]	ASD parents	(i) A self- and informant-report questionnaire of social personality, rigidity, and pragmatic language deficits (intended to parallel the 3 domains of impairment in ASD) (ii) Each question is rated on a 6-point scale, allowing a range of possible responses to maximize variability (iii) Validation has shown high sensitivity and specificity when BAPQ scores are used to predict direct clinical observations of BAP traits
	Children's Communication Checklist, 2nd Edition	Bishop, 2003 [34]	Children aged 4–16 years of age	(i) A parent-report questionnaire assessing various communication skills with age-based scaled scores and a general communication composite (ii) Scales include structural aspects of language, pragmatic language, and social relations and interests (iii) Composite scores differentiate children with communication challenges (both SLI and ASD) from typically developing children [35] (iv) ASD siblings were overrepresented in the lower range of scores compared to typically controls [22]
	Social Responsiveness Scale (SRS)	Constantino et al. 2000 [36]	Children and adults both with and without ASD	(i) An informant-report (generally parent, teacher, or spouse) questionnaire measuring autism-related traits along one continuum of social reciprocity, which purportedly reflects a single underlying vulnerability to ASD-related traits [37, 38] (ii) Widely used in many genetic studies and researched in both clinically ascertained and population-based samples [38–40] (iii) Scores from the SRS have been linked to specific areas of the genome through QTL-based analyses [41]

				(i) Assesses autism-related traits in 4 domains (social motivation, social expressivity, conversational skills, and flexibility/range of interests) via both direct clinical observation and interview
	Broader Phenotype Autism Symptom Scale (BPASS)	Dawson et al., 2007 [42]	Children and adults both with and without ASD	(ii) Adults are interviewed about themselves; parents are interviewed about their children
				(iii) Ratings for items within domains range from impaired to nonimpaired, with some questions identifying those with a level above the norm (designed to increase statistical power to detect evidence for genetic effects in QTL analyses)
				(iv) Two domains (social motivation and flexibility) have the positive QTL findings [43]
				(i) First measure developed to directly measure autism-related traits in family members via a semistructured interview and provides categorical (not continuous) information
Interviews and Direct Behavioral Observation	Family History Interview (FHI) or Family History Schedule (FHS)	Bolton, et al., 1994 [12]; Piven et al., 1997 [16]	Children and adults both with and without ASD	(ii) Informant rates of social and communication skills and range of interests of immediate and extended family members
				(iii) An algorithm determines whether there is a presence or absence of BAP traits in 3 domains: social, communication (primarily assessing a history of language/reading delays), and repetitive behaviors
				(i) A semi-structured interview measuring ASD-related personality traits
				(ii) Participants are interviewed about themselves while an informant (generally a spouse) is asked similar questions in a separate interview
	Modified Personality Assessment Schedule-Revised (M-PAS-R)	Piven et al., 1997 [15]; Piven et al., 1994 [13]	Adults	(iii) Participants are rated by trained examiners based on behavioral examples given by either the subject or the informant
				(iv) Originally including 18 domains, it was later revised to focus on 8 traits particularly applicable to ASD: conscientious, rigidity, aloof, undemonstrative, anxious, hypersensitive to criticism, unresponsive, and untactful
				(v) Rigidity and aloof traits have been the most valid and reliable discriminators [13, 15, 29].
				(i) Intended to assess verbal and nonverbal pragmatic language via 15 minutes of conversational exchange and perhaps time spent in direct assessment of parents
	Pragmatic Rating Scale (PRS)	Landa et al., 1992 [10]	Adults	(ii) Assesses skills in maintaining a flow of conversation.
				(iii) Subscales: disinhibited social communication (e.g., abrupt topic change and overly detailed descriptions), awkward/inadequate expression (e.g., vague and inadequate clarification), and odd verbal interaction (e.g., odd humor and inappropriate topics)
				(iv) Successfully differentiated ASD parents from controls in many studies (e.g., [15, 17])
